# Arthroscopic Treatment of Schatzker Type III Tibial Plateau Fractures Using a Combination of Bioabsorbable Interference Screws and Cannulated Screws

**DOI:** 10.1002/atn2.70115

**Published:** 2026-06-10

**Authors:** Renbang Huang, Zeming Li, Xifan Zheng, Jun Yao

**Affiliations:** ^1^ Orthopedics The People's Hospital of Gongcheng Yao Autonomous County Guilin China; ^2^ Department of Bone & Joint Surgery Guangxi Medical University, The First Affiliated Hospital of Guangxi Medical University Nanning China

## Abstract

Schatzker type III tibial plateau fractures, characterized by a pure depression of the lateral articular surface, predominantly affect osteoporotic, middle‐aged, and elderly patients. Successful management requires anatomical reduction and stable fixation to minimize complications like post‐traumatic arthritis. Conventional open reduction and internal fixation, which involves extensive soft‐tissue dissection and relies on fluoroscopic assessment, often fails to address concomitant intra‐articular injuries. Utilizing advancements in arthroscopy and bioabsorbable implants, we propose a technique combining arthroscopic‐assisted reduction with a composite fixation construct of bioabsorbable interference and cannulated screws. This method provides direct visualization for precise reduction, minimizes soft‐tissue trauma, and creates a biomechanically stable environment for early rehabilitation, integrating minimally invasive principles with effective fracture stabilization.

## Graphical abstract

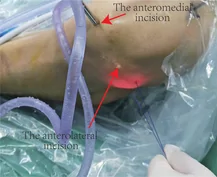

**VIDEO 1:** The anterolateral portal was initially used as the viewing portal. Arthroscopic examination revealed a tear in the body of the lateral meniscus and a depressed fracture of the central articular surface of the lateral tibial plateau. The viewing portal was then switched to the anteromedial portal, while the anterolateral portal was used for instrumentation. A polydioxanone suture II suture was introduced through a site approximately 1.5 cm adjacent to the standard anterolateral portal. It was looped around and used to retract the anterior horn of the lateral meniscus, thereby adequately exposing the depressed fracture fragment. An anterior cruciate ligament tibial aimer was inserted through the anterolateral portal, set at a guide angle of 47°, and positioned over the posterior one‐third of the depressed area. A 1.2 mm K‐wire was drilled into the joint cavity through the aimer. A 7.0 mm cannulated reamer was then used to create a bone tunnel to a depth of approximately 2.0 cm along the K‐wire. The same procedure was repeated for the anterior one‐third of the depression, involving K‐wire insertion and reaming. A bioabsorbable interference screw was slowly inserted sequentially from posterior to anterior into the prepared tunnels. Under arthroscopic visualization, the depressed posterior fragment was gradually elevated by the radial expansion force of the screw until it was flush with the surrounding articular surface. Subsequently, under intraoperative fluoroscopic guidance, a 1.2 mm K‐wire was drilled as a guide pin parallel to the joint line, approximately 1.0 cm inferior to the lateral rim of the tibial plateau. A 2.7 mm cannulated drill was used for reaming, followed by the insertion of a cannulated compression screw over the 1.2 mm guide pin. This created a composite fixation construct, enhancing the overall stability. Repeat fluoroscopy confirmed maintained fracture reduction and appropriate implant position. Finally, the knee was taken through a passive range of motion, which showed stable fixation without fragment displacement. Final arthroscopic inspection confirmed a continuous and congruent articular surface. The joint was irrigated, all instruments were removed, and the portals were closed in layers. Video content can be viewed at https://doi.org/10.1002/atn2.70115.

Tibial plateau fractures represent a common type of intra‐articular fracture, whose treatment outcomes directly influence knee joint function. Among the Schatzker classification of tibial plateau fractures, types II and III are the most common.^1^, ^2^ Schatzker type III fractures are characterized by a pure depression of the central articular surface of the lateral tibial plateau, and typically occur in middle‐aged and elderly osteoporotic patients following low‐energy axial loading.^3^ If this type of fracture is not treated promptly, or if anatomical reduction and rigid internal fixation are not achieved during treatment, patients are prone to developing secondary complications such as post‐traumatic arthritis and joint stiffness, which severely impair their quality of life and daily work capacity.^3^, ^4^ Surgical treatments for tibial plateau fractures are diverse. Currently, common surgical procedures for Schatzker type III fractures include traditional open reduction and internal fixation (ORIF), percutaneous pry reduction and internal fixation, balloon tibioplasty, and arthroscopically assisted reduction and internal fixation.^5^, ^6^, ^7^, ^8^ However, the aforementioned three surgical approaches are often limited in their utility due to the lack of direct visualization for assessing articular reduction and the inability to address concomitant intra‐articular injuries. In terms of fixation materials, options commonly employed in clinical practice include cannulated screws alone, buttress plates, bone grafting, or bone cement augmentation.^1^, ^3^, ^6^, ^9^, ^10^ Therefore, we propose the combined use of bioabsorbable interface screws and cannulated screws. Through the compressive effect of the interface screws, the anchoring force of the cannulated screws is enhanced, thereby constructing a composite fixation construct with superior biomechanical properties. This article aims to systematically elaborate on the surgical methods and key technical points of using absorbable interface screws combined with cannulated screws for the treatment of Schatzker type III tibial plateau fractures under arthroscopic assistance, in order to provide a more reliable and minimally invasive treatment option for clinical practice.

## SURGICAL TECHNIQUE

**FIGURE 1:**
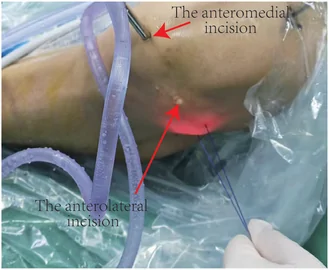
Surgical incisions and patient positioning. Standard anteromedial and anterolateral arthroscopic portals were established. The anterolateral portal served as the viewing portal, and the anteromedial portal was used as the working portal. The affected limb was positioned in the “figure‐of‐4” position (hip and knee flexion, abduction, and external rotation). A shaver system (STAR, BB01SF, 4.0 × 193 mm, Beijing, China) was introduced via the anteromedial portal to evacuate hemarthrosis and debride fragmented tissue from the joint cavity. Patient side and position: left knee.

**FIGURE 2:**
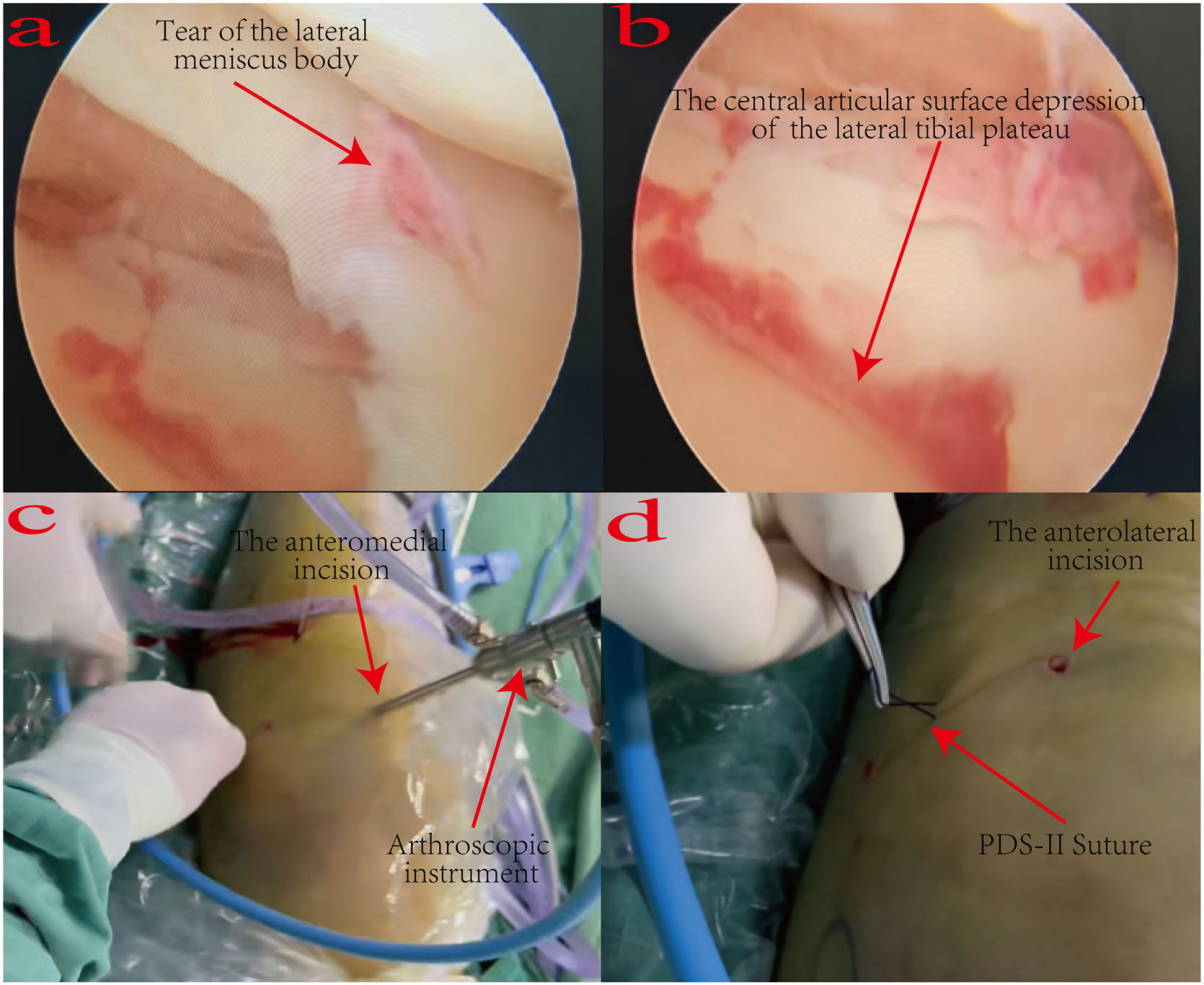
Arthroscopic evaluation and exposure of the fracture area. The anterolateral incision was used as the viewing portal. (a) A tear in the body of the lateral meniscus was identified. (b) Depression of the central articular surface of the lateral tibial plateau was observed, with the fracture area measuring approximately 3 × 4 cm. The depression was approximately 6 mm anteriorly and 3 mm posteriorly. (c,d) The viewing portal was switched to the anteromedial portal. A PDS‐II suture was introduced through a site approximately 1.5 cm adjacent to the anterolateral portal, looped around, and used to retract the anterior horn of the lateral meniscus to expose the fracture area. Patient side and position: left knee.

**FIGURE 3:**
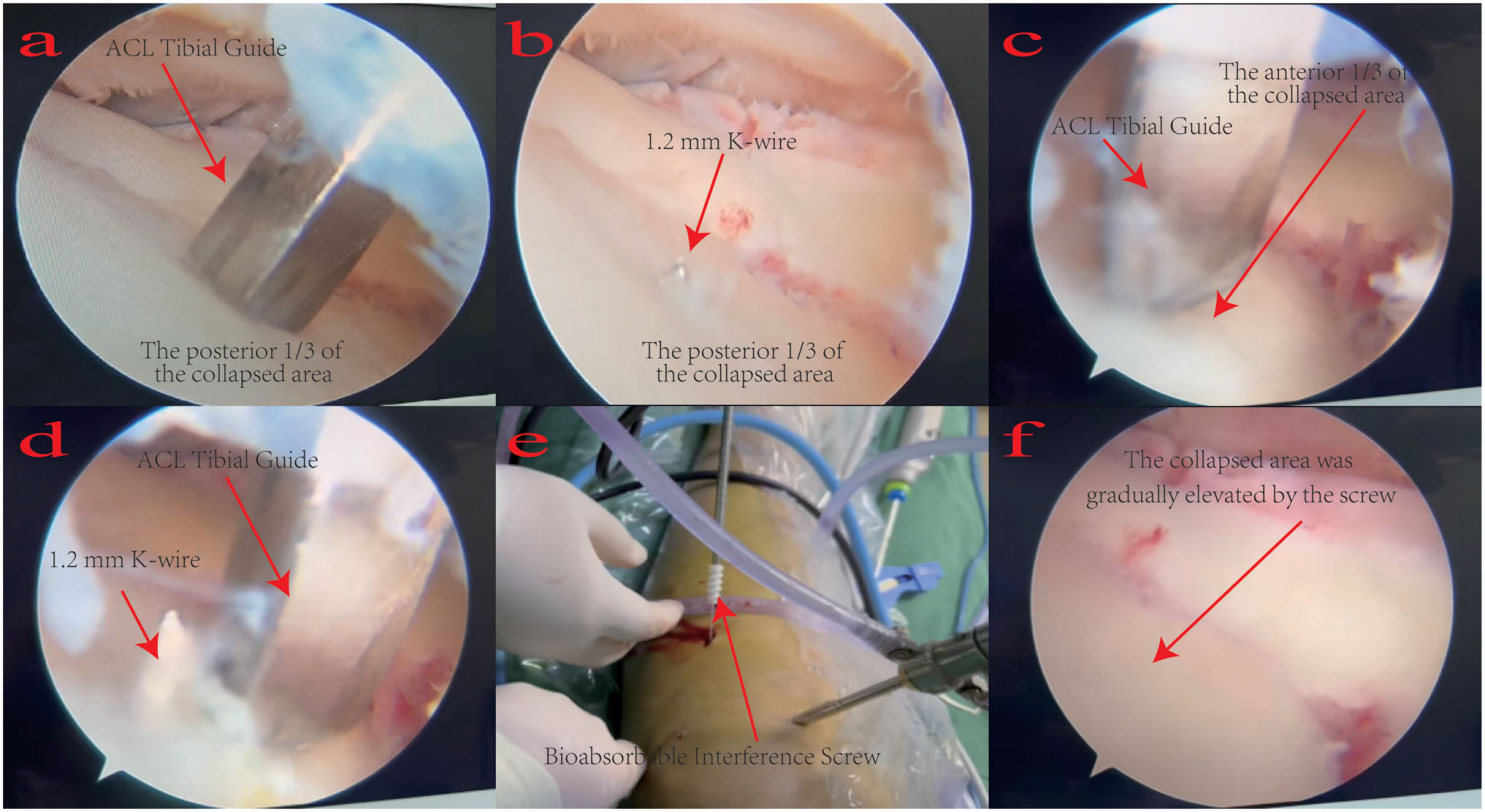
Inserted bioabsorbable interference screw: external and arthroscopic views. The anteromedial incision was used as the observation approach, and the anterolateral incision as the operational approach. (a,b) Anterior cruciate ligament (ACL) tibial locator (STAR) was set with a guide angle of 47°. The locator was positioned at the posterior 1/3 of the collapsed area. A 1.2 mm Kirschner wire was drilled into the joint cavity along the guide, followed by reaming the hole with a 7.0 mm tibial drill along the wire to a length of approximately 2.0 cm. (c,d) ACL tibial locator (STAR) was set with a guide angle of 47°. The locator was positioned at the anterior 1/3 of the collapsed area. A 1.2 mm Kirschner wire was drilled into the joint cavity along the guide, followed by reaming the hole with a 7.0 mm tibial drill along the wire to a length of approximately 2.0 cm. (e) An absorbable interference screw (STAR, AIS‐1, 8 × 23 mm) was screwed into the created bone tunnel along the 1.2 mm Kirschner wire to compress and push the collapsed bone fragment. (f) Under arthroscopy, the fractured fragment in the collapsed area was gradually elevated by the screw compression until it was flush with the surrounding articular surface. Patient side and position: left knee.

**FIGURE 4:**
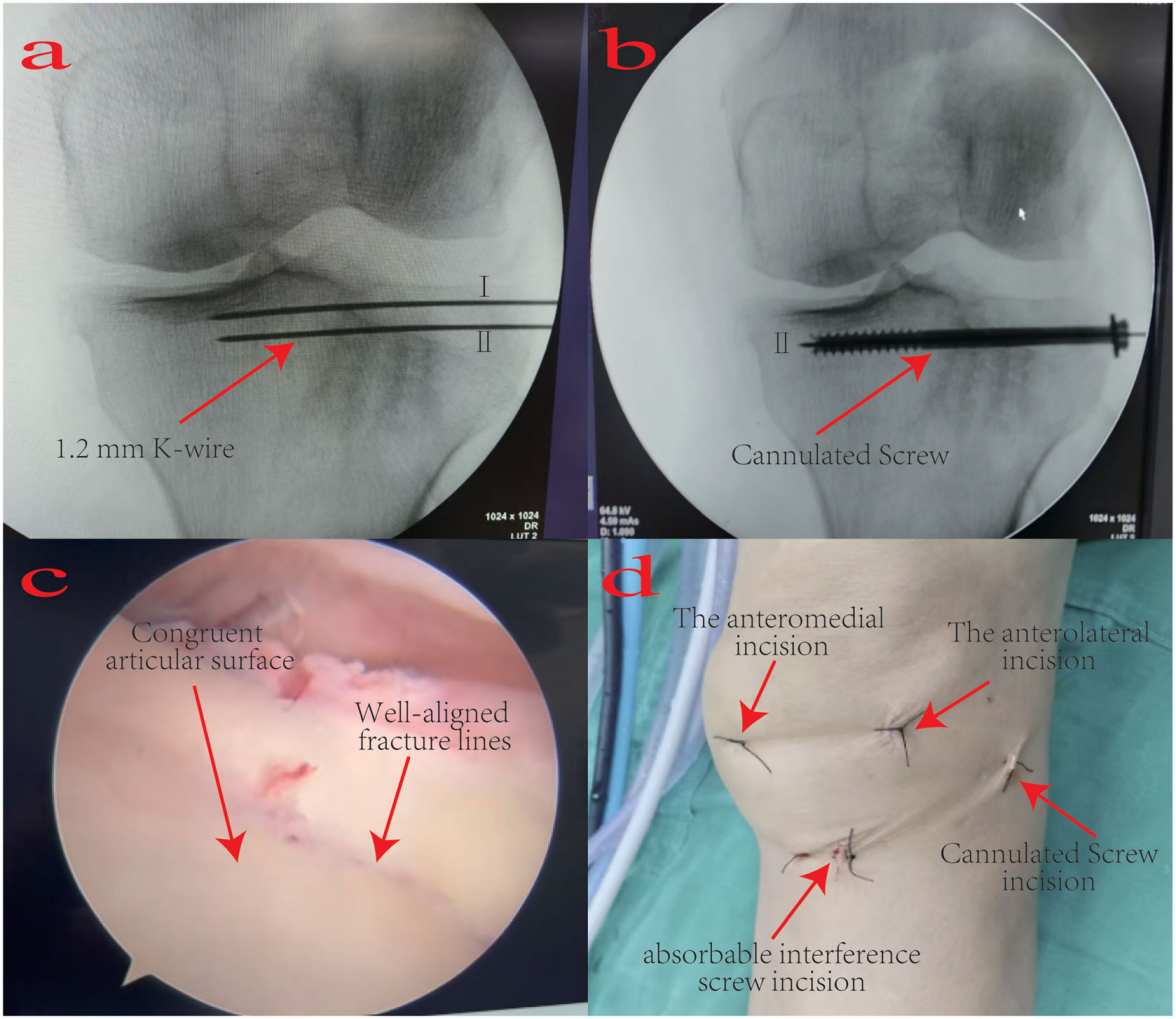
Insert a cannulated screw and Final arthroscopic inspection. (a) A 1.2 mm K‐wire was drilled as a guide pin (П) parallel to the joint line, approximately 1.0 cm inferior to the lateral rim of the tibial plateau. After fluoroscopy confirmed satisfactory positioning, a 2.7 mm cannulated drill was used for reaming, followed by insertion of a cannulated compression screw (Dabo Medical, GJYD VII, 3.5 × 42 mm) over the guidewire. This construct, combined with the two underlying bioabsorbable interference screws, formed a composite fixation system that enhanced overall stability. (b) Fluoroscopy showed maintained fracture reduction and appropriate implant position. (c) After passive knee motion, arthroscopic reevaluation confirmed a continuous and congruent articular surface. (d) Appearance of the surgical wound after closure. Patient side and position: left knee.

The patient is placed in the standard supine position. Following satisfactory continuous epidural anesthesia, a pneumatic tourniquet is applied to the proximal thigh of the affected limb and inflated. Routine antiseptic skin preparation and draping are performed. Step 1: Standard anteromedial and anterolateral arthroscopic portals are established under aseptic technique (Figure 1, Video 1). The anterolateral portal serves as the viewing portal, and the anteromedial portal is used as the working portal. A shaver system (STAR, BB01SF, 4.0 × 193 mm, Beijing, China) is introduced via the anteromedial portal to evacuate hemarthrosis and debris from the joint cavity. A systematic arthroscopic examination is performed, sequentially inspecting the suprapatellar pouch, medial and lateral gutters, anterior and posterior cruciate ligaments, and the medial and lateral menisci, which confirm the absence of concomitant injuries. Step 2: The affected limb is positioned in the “figure‐of‐4” position (hip and knee flexion, abduction, and external rotation) (Figure 1) to facilitate access to the lateral compartment. Arthroscopic evaluation reveals a tear in the body of the lateral meniscus (Figure 2a) and a depressed fracture of the central aspect of the lateral tibial plateau. The fracture measures approximately 3 cm × 4 cm, with approximately 6 mm of depression anteriorly and 3 mm posteriorly (Figure 2b). To optimize exposure of the fracture site, the viewing portal is switched to the anteromedial portal. A spinal needle is introduced approximately 1.5 cm adjacent to the standard anterolateral portal, through which a polydioxanone suture II  suture (Johnson & Johnson, Edinburgh, Scotland) is passed to loop around and retract the anterior horn of the lateral meniscus, thereby effectively exposing the depressed fracture fragment (Figure 2c,d). An anterior cruciate ligament (ACL) tibial aimer (STAR, Beijing, China) is then introduced through the anterolateral portal, set at a 47° angle, and positioned over the posterior one‐third of the depressed area (Figure 3a). A 1.2 mm K‐wire is drilled into the joint cavity through the guide. A 7.0 mm cannulated reamer is used to create a bone tunnel to a depth of approximately 2.0 cm along the guidewire, with arthroscopic visualization maintained to prevent iatrogenic chondral damage (Figure 3b). The same procedure is repeated for the anterior one‐third of the depressed area, involving guidewire insertion and reaming (Figure 3c,d). Subsequently, a bioabsorbable interference screw (STAR, AIS‐1, 8 × 23 mm, Beijing, China) is inserted sequentially from posterior to anterior into the prepared tunnels (Figure 3e). Under direct arthroscopic vision, the depressed posterior fragment is gradually elevated by the radial expansion force of the screw until it is flush with the surrounding articular surface, achieving anatomical reduction of the joint line (Figure 3f). Step 3: A 1.2 mm K‐wire is inserted as a guidewire parallel to the joint line, approximately 1.0 cm inferior to the lateral tibial plateau rim. Its position is confirmed under fluoroscopy (Figure 4a). After drilling with a 2.7 mm cannulated drill (Dabo Medical, Xiamen, China), a cannulated compression screw (Dabo Medical, GJYD VII, 3.5 × 42 mm, Xiamen, China) is inserted over the guidewire, creating a composite fixation construct that enhanced overall stability (Figure 4b). Postoperative fluoroscopy confirms maintained fracture reduction and appropriate implant position. Step 4: The knee is taken through a passive range of motion, showing stable fixation without fragment displacement. Final arthroscopic inspection confirms a continuous and congruent articular surface (Figure 4c). The joint is irrigated, instruments are removed, and the portals are closed in layers (Figure 4d). The procedure is concluded.

## DISCUSSION

The defining feature of a Schatzker type III fracture is a central depression confined to the load‐bearing area of the lateral tibial plateau.^3^, ^11^ The core of treatment lies in achieving anatomical reduction of the articular surface and obtaining stable and rigid initial fixation, thereby laying a foundation for early functional exercise and minimizing the occurrence of complications such as post‐traumatic arthritis and joint stiffness.^12^, ^13^ Although ORIF provides rigid support, the extensive soft tissue dissection required increases the risk of complications such as impaired wound healing, infection, and devitalization of bone fragments.^14^, ^15^, ^16^ Moreover, it does not allow for direct visual assessment of the articular reduction.^11^ Although percutaneous prizing reduction has the advantage of minimal invasiveness, it also has drawbacks such as “blind” manipulation, great difficulty in percutaneous elevation of collapsed bone fragments, reliance on fluoroscopy for evaluating articular surface reduction, and inability to effectively manage intra‐articular combined injuries.^7^, ^17^ Thus, the development of techniques aimed at achieving both minimally invasive anatomical reduction and rigidly stable fixation has emerged as a significant goal for orthopaedic surgeons.

With the development of arthroscopic techniques and internal fixation materials, new surgical concepts have been brought to the treatment of Schatzker type III fractures. As shown in the surgical steps of this study, arthroscopy provides unmatched intra‐articular visualization, and its advantages are absolute. Among them, the “Pearls and Pitfalls” of this technology are the aspects that we need to pay attention to (Table 1). Firstly, it allows surgeons to achieve anatomical reduction of the articular surface under direct arthroscopic visualization and monitoring, enabling immediate and accurate assessment of the flatness of the articular surface—an advantage that cannot be matched by intraoperative X‐ray fluoroscopy. Secondly, it enables comprehensive exploration and management of structures such as menisci and ligaments, effectively avoiding the omission of intra‐articular combined injuries that may occur in traditional open surgery.^18^, ^19^ In this case series, we modified the exposure technique by retracting the anterior horn of the lateral meniscus using a polydioxanone suture II suture, which clearly revealed the obscured depressed fracture fragment and provided a foundation for subsequent precise positioning and direct visual reduction. Compared with the extensive soft tissue dissection required in traditional open surgery, this technique only requires several minimal incisions, significantly reducing surgical trauma and bleeding, preserving the periarticular blood supply around the tibial plateau, and creating favorable conditions for fracture healing. For Schatzker type III fractures, a stable and reliable fixation method is critical to preventing early postoperative reduction failure. Although standalone cannulated screw support is a common approach, its holding capacity often proves insufficient in osteoporotic patients with significant bone defects. This study employed a composite fixation technique combining a bioabsorbable interference screw with cannulated screws. Its biomechanical advantages lie in the following: The bioabsorbable interference screw offers ideal initial mechanical strength and excellent biocompatibility. Its radial expansion effect within the bone tunnel directly acts on the depressed fracture fragment, achieving precise “point‐to‐point” elevation and restoring articular congruity.^20^, ^21^ During the procedure, screws were inserted at two points—anterior and posterior to the depressed zone—allowing gradual elevation of the fracture fragment until it was flush with the surrounding articular surface. In contrast to the “passive elevation” achieved by conventional reduction instruments, this technique provides a more controlled reduction with closer bony apposition. The interference fit between the screw and the bone interface effectively resists redisplacement. Subsequently, a cannulated compression screw inserted parallel to the joint line further constructs a composite fixation system characterized by vertical support and transverse compression, significantly enhancing overall stability. This design compensates for the limitations of standalone cannulated screws—namely, insufficient torsional resistance and limited anchoring strength—thereby effectively reducing the risk of early postoperative loss of reduction. Passive knee motion performed before wound closure confirmed the absence of fragment displacement, validating the immediate stability of the composite construct. Furthermore, the bioabsorbable interference screw obviates the need for a second surgery for hardware removal, avoiding additional trauma and health care burden. Its degradation profile aligns with the bone remodeling process, adhering to the principles of biological fixation. As observed intraoperatively, the depressed articular surface was synchronously elevated during the insertion of the bioabsorbable interference screw, confirming that this fixation method provides sufficient initial support strength. The subsequent addition of a cannulated compression screw, placed parallel to the joint line, further reinforced the support structure. Together, they provide the mechanical stability required for early nonweight‐bearing knee mobilization in the postoperative period.

**TABLE 1:** Pearls and Pitfalls

Pearls	Pitfalls
Arthroscopy provides direct intra‐articular visualization, allowing for anatomic reduction of the articular surface under direct vision. This represents an advantage unmatched by intraoperative fluoroscopy.	In patients with severely comminuted fractures or significant osteoporosis, the holding power of the interference screw is significantly reduced, necessitating cautious patient selection.
Retraction of the anterior horn of the lateral meniscus using a polydioxanone suture II suture provides clear exposure of the depressed fracture fragment, enabling subsequent precise positioning and facilitating reduction under direct visualization.	The technique is heavily dependent on a specialized arthroscopic system and specific targeting instruments. Although the initial strength of the bioabsorbable screw is sufficient, its strength gradually diminishes during the degradation process.
The depressed area was conceptually divided into anterior, middle, and posterior thirds. The bioabsorbable interference screws were then inserted sequentially, first into the posterior third and then into the anterior third of the zone.	Close proximity of the screw tunnels must be avoided to prevent interference between the implants, which risks compromising both the restoration of articular congruity and the mechanical strength of the composite construct.
During creation of the bone tunnel, excessive depth must be avoided to prevent violation of the subchondral plate; sufficient bone stock must be preserved to allow radial expansion of the screw for fragment elevation; and iatrogenic damage to the articular cartilage must be meticulously prevented.	When placing cannulated screws in the lateral tibial plateau, intra‐articular penetration due to suboptimal fluoroscopic guidance must be carefully avoided. Meticulous care must also be exercised to avoid injury to the laterally situated common peroneal nerve.
The technique requires only a few minimal incisions, which significantly reduces surgical trauma, preserves the periarticular blood supply around the tibial plateau, and creates favorable conditions for fracture healing.	A small subset of patients may develop an aseptic inflammatory reaction to the bioabsorbable material, leading to postoperative discomfort.

Although this technique has numerous advantages, it also has certain limitations (Table 2) Currently, this technique is only applicable to Schatzker type III fractures with simple central collapse of the lateral plateau and no severe comminuted fractures. Its applicability may be limited for complex fractures. In patients with severe osteoporosis, the compressive anchoring force of absorbable interface compression screws is significantly reduced; even when combined with cannulated screws, there remains a risk of reduction loss and internal fixation loosening, so cautious selection is required. This technique demands surgeons to have extensive arthroscopic operation experience, making it difficult to implement in primary hospitals or medical institutions lacking arthroscopic equipment, which limits its popularization and application. Compared with traditional open surgery, this technique has a longer learning curve than open reduction or percutaneous prizing reduction. It is highly dependent on equipment and instruments, requiring specialized arthroscopic systems and positioning devices. Although the initial strength of absorbable screws is sufficient, it is always lower than that of metal screws. In the early postoperative period, their strength gradually decreases with the degradation process. If bone healing is delayed while the screw strength has significantly decreased, there is a risk of late collapse. There are potential foreign body reactions: a very small number of patients may develop aseptic inflammatory reactions to absorbable materials, leading to postoperative discomfort. There is a lack of long‐term, large‐sample evidence‐based medical evidence to confirm its advantages over traditional methods in delaying post‐traumatic arthritis and improving long‐term knee joint function.

**TABLE 2:** Advantages and Disadvantages

**Advantages**	**Disadvantages**
The minimally invasive arthroscopic approach uses small portals and limited soft‐tissue dissection, preserving the periarticular envelope and meniscus and reducing the risk of wound complications and soft‐tissue morbidity.	The indication spectrum is relatively narrow; the technique is best suited for localized lateral plateau depression with an intact cortical rim, whereas severely comminuted bicondylar fractures or marked malalignment often require open reduction and plate fixation.
Direct arthroscopic visualization of the articular surface allows precise assessment and stepwise adjustment of the reduction, improving joint congruity and decreasing the likelihood of residual step‐off that may be missed on fluoroscopy alone.	The procedure is technically demanding with a steep learning curve; inaccurate tunnel or screw placement and iatrogenic cartilage or meniscal injury may occur in inexperienced hands.
Arthroscopy permits comprehensive inspection and simultaneous management of concomitant intra‐articular lesions (e.g., meniscal tears, chondral damage, cruciate‐ligament pathology), thereby lowering the risk of missed injuries.	Elevation of the depressed fragment with limited subchondral support may be less reliable in severe osteoporotic bone or in large defects, potentially increasing the risk of secondary subsidence compared with open elevation, structural bone grafting and buttress plating.
Subchondral elevation using bioabsorbable interface screws restores the joint surface while minimizing long‐term hardware prominence or irritation adjacent to the articular cartilage.	This technique relies heavily on arthroscopic equipment and specialized targeting instruments.
Reduced soft‐tissue trauma and stable internal fixation facilitate earlier knee mobilization, less postoperative pain and potentially faster functional recovery compared with conventional open approaches.	Long‐term, large‐sample comparative data versus traditional open plating are still limited, and the durability of reduction and long‐term clinical outcomes, particularly in high‐energy or osteoporotic fractures, remain to be fully established.

In summary, we conclude that the technique of arthroscopically assisted bioabsorbable interference screw combined with cannulated screw fixation not only achieves minimally invasive intervention and direct visual anatomical reduction of the articular surface but also maximally preserves soft tissue and osseous vascularity. Furthermore, by means of a biomechanically superior composite fixation construct, it effectively circumvents multiple limitations inherent to conventional treatments. This technique significantly reduces the risk of postoperative complications and holds substantial clinical importance for improving knee function and quality of life in patients. It merits consideration as a preferred treatment option for Schatzker type III tibial plateau fractures in clinical practice.

## DISCLOSURES

The authors (R.H., Z.L., X.Z., J.Y.) declare that they have no known competing financial interests or personal relationships that could have appeared to influence the work reported in this article.
